# A novel hedgehog inhibitor iG2 suppresses tumorigenesis by impairing self‐renewal in human bladder cancer

**DOI:** 10.1002/cam4.802

**Published:** 2016-07-27

**Authors:** Lihong Zhu, Chen Ni, Baijun Dong, Yong Zhang, Yuefeng Shi, Haitao Niu, Chong Li

**Affiliations:** ^1^ Institute of Plant Protection and MicrobiologyZhejiang Academy of Agricultural SciencesHangzhou310021China; ^2^ Medical Research Centerthe First Affiliated Hospital of Zhengzhou UniversityZhengzhou450052China; ^3^ Department of UrologyRenji HospitalSchool of MedicineShanghai Jiao Tong UniversityShanghai200127China; ^4^ Department of Urologythe Affiliated Hospital of Qingdao UniversityQingdao266003China; ^5^ Laboratory Animal CenterCAS Key Laboratory of Infection and ImmunityInstitute of BiophysicsChinese Academy of SciencesBeijing100101China

**Keywords:** Bladder cancer, Gli2, hedgehog, iG2

## Abstract

Tumor recurrence is still a major challenge for clinical treatment of bladder cancer. Cumulative evidences indicate cancer stem cells (CSCs) contribute to drug resistance and leave a putative source for disease relapse. Identifying novel agents targeting CSCs may represent a new paradigm in the therapy of bladder cancer. Here, we separated a novel hedgehog (Hh) inhibitor, iG2, from s*treptomyces roseofulvus*, which dramatically blocked the activation of Gli2 in bladder cancer cells. The iG2 strongly repressed the growth of cancer cells rather than the peri‐tumor stroma cells. Attenuated proliferation and enhanced apoptosis of tumor cells were observed upon iG2 stimulation. Furthermore, iG2 reduced the self‐renewal ability of bladder CSCs as well as the tumor formation. Collectively, iG2 is potentially used as a novel therapeutic agent for bladder cancer by targeting self‐renewal through inhibiting Hh pathway.

## Introduction

Emerging evidence suggests that cancer stem cells (CSCs) contribute to the progression of bladder cancer 1, 2, 3. Bladder cancer is a heterogenerous disease, with 70% of patients presenting with superficial tumors and 30% presenting as muscle‐invasive disease 4, 5. The superficial tumor is generally not life threatening but tends to recur after treatment, and progresses to invasive disease which is associated with a high risk of death from metastases 5. In human primary bladder cancer, subpopulations of CSCs have been identified and characterized, largely contributing to the invasive disease of bladder cancer 2, 3, 6. CSCs exhibit stem cell properties of self‐renewal and differentiation, which are uniquely capable of initiating and sustaining tumor growth 7. Quiescent CSCs may evade of cell death, survive therapeutic intervention and result in recurrence 8, 9, 10, 11, 12, 13. Targeting CSCs may achieve durable remission in the treatment of bladder cancer 2, 14.

The hedgehog (Hh) pathway is one of the core regulatory genes for CSCs, which is stronghly linked to self‐renewal 15, 16. Hh signaling regulates the proliferation, migration, and differentiation of target cells during embryonic development in a spatial, temporal, and concentration‐dependent manner 17. Pathway activation is initiated by binding of Hh ligand to Patched (Ptch1) which constitutively represses the activity of Smoothened (Smo). Following Hh ligand binding to Ptch, the repression of Smo is released, and the three Gli transcription factors are modulated 18. Gli1 acts as a transcriptional activator and Gli3 as a repressor, whereas Gli2 can either activate or repress gene expression depending on posttranscriptional and posttranslational modifications 19. The balance between the activating and repressive forms of the Glis results in the expression of target genes, including Ptch1, Gli1, and Jag2 20, 21. In the context of bladder cancer, mutations of Smo and persistent activation of Gli have been observed 2, 22, 23. On injury, sonic hedgehog (Shh)‐expressing basal cells include stem cells in urinary bladder are capable of regenerating all cell types within the urothelium 24. Moreover, muscle‐invasive bladder carcinomas arise exclusively from these Shh‐expressing stem cells in basal urothelium 1. Therefore, Hh pathway provides a potential target in bladder cancer to abate the self‐renewal of CSCs.

In clinical treatment of cancer patients, inhibitors of Hh have received beneficial responses in many kinds of cancers 23, 25. Hh inhibitors block both intrinsic signaling in cancer cells and extrinsic signaling in stroma cells to decrease tumor growth 26. Currently, most of Hh inhibitors are developed to target SMO 15, 27. But, there is a clear need for inhibitors that act downstream of SMO, such as Gli inhibitors to overcome SMO resistance 27, 28, 29. In bladder cancer, Gli2 inhibition by GANT61 has been shown to reduce cell invasiveness 30. However, whether Hh inhibitors can be used for the treatment of bladder cancer is still unknown.

Here, we isolated a novel Hh inhibitor iG2 from s*treptomyces roseofulvus*, which dramatically blocked the activation of Gli2 in bladder cancer cells. The results demonstrate that iG2 impairs self‐renewal of CSCs to suppress tumorigenesis of bladder cancer. Thus, iG2 may be used as a novel therapeutic agent for bladder cancer in clinic.

## Materials and Methods

### Separation of iG2

Streptomyces roseoflavus subsp. Hangzhouensis n. subsp. was separated from the soil sampled in the suburb of Hangzhou, China. After leaching mycelia, the fermentation liquor was extracted, concentrated, and put on silica gel H column and eluted with ethyl acetate: ethylene dichloride (1:2) buffer. The enriched portion was separated again with ethyl acetate: ethylene dichloride (1:8) buffer.

### Xenograft model

The cancer tissues were transported from the operating theater immediately, minced and implanted subcutaneously (s.c.) plus with Matirgel (BD) through small incisions in NOD/SCID mice under general anesthesia.

### Cell culture

Bladder cancer and peri‐tumor tissues were minced and digested with collagenase IV (GIBCO) at 37°C for 2 h. The cell suspension was filtered and stained with antibodies to human CD45 (340934, BD) and CD31 (340297, BD). Then the cells were sorted into a 6‐well plate using a FACSArilaII flow cytometry system (BD Biosciences, Bedford, Ohio, USA) and cultured with RPMI‐1640 medium.

### [^3^H]‐TdR incorporation assay

Bladder cancer cells were seeded on 96‐well culture plates, cultured until the cells reached 70–80% confluency, serum starved in RPMI‐1640 for 24 h, stimulated with iG2 or PBS for 72 h, pulsed with [^3^H]‐thymidine for 4 h and then their [^3^H]‐thymidine incorporation was measured in the liquid scintillation counter LKB1219.

### Western blotting

Bladder cancer cells were treated with iG2 for 24 h. Whole‐cell proteins were extracted and separated with SDS‐PAGE before transfer onto a nitrocellulose membrane. Membranes were blocked and then probed with primary antibodies and HRP‐conjugated secondary antibodies (Sigma‐Aldrich, St. Louis, Missouri, USA).

### UTR–mediated luciferase reporter expression and cytotoxicity measurements

HEK293 cells were transfected with a GEMS reporter vector containing the luciferase open‐reading frame and under posttranscriptional control of the Gli2 5′ and 3′ UTRs, treated with iG2 or vehicle control overnight, and then luciferase reporter activity and cytotoxicity were determined using Bright‐Glo and CellTiter‐Glo assays (Promega, Madison, Wisconsin, USA), respectively.

### IC_50_ determination

Bladder cancer cells were seeded in 96‐well plates and treated with various concentrations of iG2 dissolved in 1% FBS medium for 24 h. IC50 was calculated using Cell Counting Kit (CCK‐8) (Sigma, Darmstadt, Germany).

### Apoptosis assay

IG2‐ or PBS‐treated primary bladder cancer cells were stained with an Annexin V‐FITC kit (Sigma‐Aldrich). Cells were washed, digested, collected, resuspended, stained with annexin V‐FITC and propidium iodide (BioVision, Milpitas, CA), incubated for 10 min at room temperature in the dark. Annexin V‐positive cells were analyzed with a FACSCalibur flow cytometer (BD Biosciences).

### Cell attachment assay

Cell‐substrate attachment assays were performed using a modification of the method described 31. Briefly, 96‐well plates were incubated with matrigel (BD Biosciences) and blocked with heat‐denatured BSA (Sigma) for 1 h. Near‐confluent cells were harvested with trypsin (Invitrogen, Carlsbad, California, USA) and resuspended in RPMI‐1640 with 1% FBS (Invitrogen), and recovered at 37°C for 15 min. Attached cells were fixed and stained with crystal violet (Sigma). The absorbance of each well was measured at 575 nm with an ELISA reader.

### Cell migration assay

This assay was performed with a modified Boyden chamber (8 *μ*m pore size; Costar, Corning, New York, USA). The transwells were coated with matrigel solution and then blocked with 1% BSA in PBS. Tumor cells were trypsinized, washed, and resuspended in serum‐free medium containing 1% BSA. After incubation for 4 h at 37°C, cells remaining at the upper surface of the membrane were removed, whereas the cells migrating to the lower surface were fixed with ethanol and stained with Giemsa solution.

### Colony formation assay

Bladder cancer cells were suspended with soft agar culture media into six‐well plates at a density of 1000 cells/well. After 2–3 weeks, colonies (≥10 cells) were counted and photographed.

### Sphere‐formation assay

Single bladder cancer cells were seeded into six‐well plates with ultralow attachment surfaces (Corning, Corning, NY). Cells were cultured in DMEM/F12 media (Gibco, Waltham, MA) supplemented with bFGF, EGF, N2, B27 (Invitrogen), and streptomycin (Gibco). Sphere number was calculated 2 weeks later.

### Silencing of Gli2

The RNA sequence against Gli2 for RNAi were designed based on pSUPER system instructions (Oligoengine) and cloned into pSUPER‐puro that expresses 19 nt hairpin‐type short hairpin RNA (shRNA) with a 9 nt loop. Gli2 shRNA‐encoding sequences were as follows: 5′‐ GCTGACGTGTGCAGTAATACT‐3′. The inserted shRNAs (pSUPER‐shGli2) were confirmed by DNA sequencing. Bladder cancer cells were transfected with Lipofectamin 2000 (Invitrogen). Gli2 silenced cells were selected with puromycin (Sigma).

### BrdU and Ki‐67analysis

BrdU was added into nonadherent spheres. After 4 h, cells were fixed, permeabilized, DNase treated and stained with anti‐BrdU antibody (BD Pharmingen). For Ki67 (51‐36525X, BD) analyses, cells were fixed and permeabilized before the intracellular stain. Cells were analyzed using the BD LSR II or FACSCanto II flow cytometers.

## Results

### The iG2 blocked Hh pathway in bladder cancer cells

**Figure 1:**
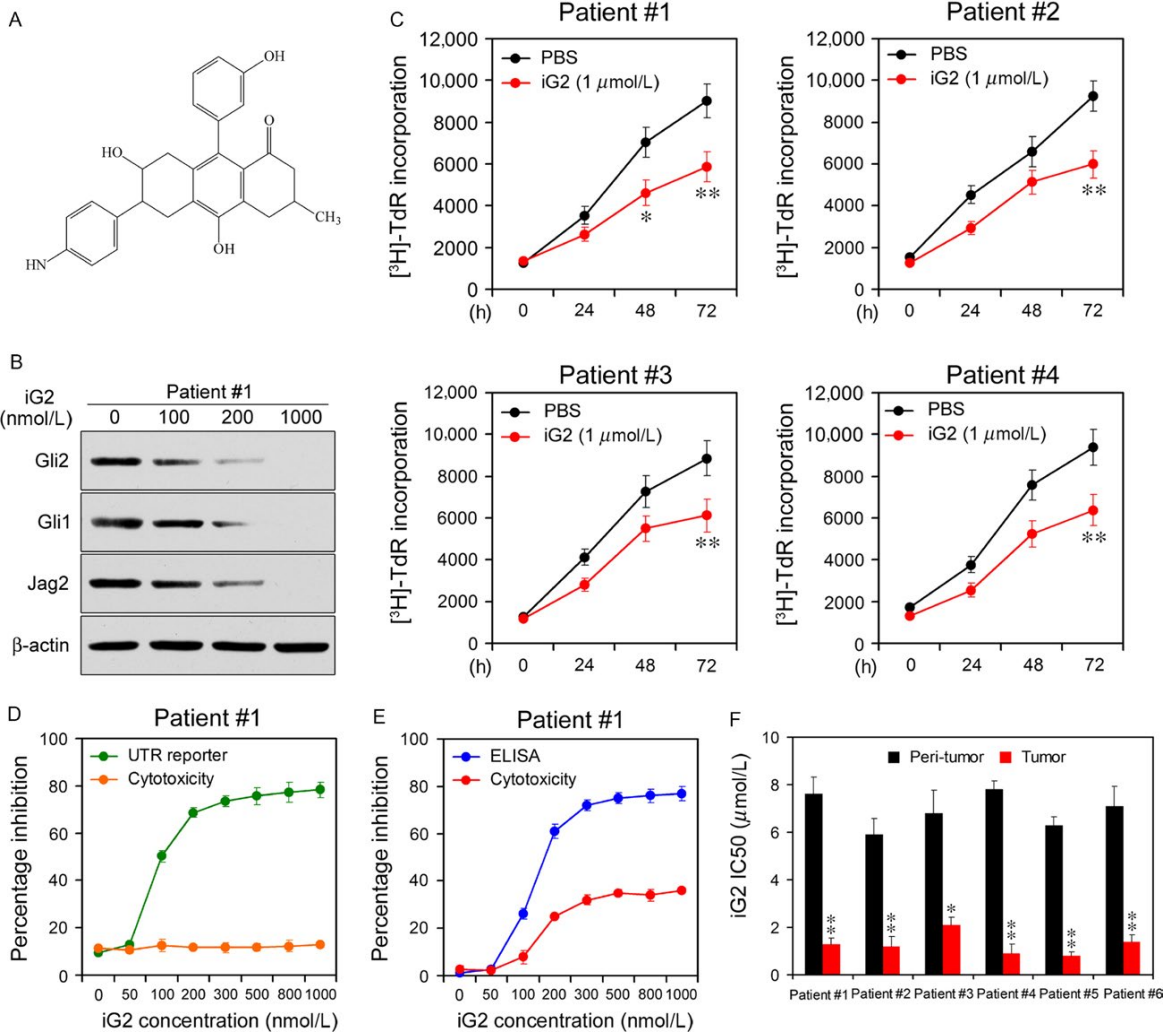
Inhibition of Hh pathway by iG2 in bladder cancer cells. (A) The chemical structure of iG2. (B) Inhibition of Gli2, Gli1 and Jag2 by iG2. Tumor cells from patient 1# were stimulated with iG2 at indicated concentration for 24 h. The expression of Gli2, Gli1 and Jag2 in whole protein lysates was determined by western blot. (C) Inhibition of the colorectal cancer cell growth in vitro by iG2. Tumor cells were separated from four cancer patients and stimulated with iG2 (1 M) for indicated time. The incorporation of [^3^H]‐TdR into tumor cells was examined. (D) Dose‐dependent inhibition of UTR‐mediated luciferase Gli2 reporter expression and cytotoxicity measurements in HEK293 cells. Data are expressed as mean ± SD of *n* = 3 replicates. (E) Gli2 inhibition (as measured by ELISA) and cytotoxicity in T24 cells following iG2 treatment. Data are expressed as mean ± SD of *n* = 3 replicates. (F) IC_50_ of iG2 as to cancer cells and adjacent nontumor cells from six patients.

A novel agent iG2, which inhibited Hh pathway, was isolated from s*treptomyces roseofulvus*. The result showed that iG2 strongly inhibited the expression of Hh pathway transcriptional factors Gli1 and Gli2, as well as the downstream protein Jag2 in primary bladder cancer cells (Fig. 1A and B). When iG2 was added into the culture medium, the growth of bladder cancer cells from four patients was all repressed (Fig. 1C). Next, the cytotoxicity of iG2 to bladder cancer cells and peri‐tumor stroma cells was examined. Subsequent characterization demonstrated that iG2 inhibited not only the UTR‐mediated reporter expression (Fig. 1D) but also endogenous Gli2 expression in human bladder cancer T24 (Fig. 1E). Moreover, the IC50 of iG2 to peri‐tumor stroma cells was generally higher than that to tumor cells (Fig. 1F). These results suggest that iG2 is a putative inhibitor of Gli2 and more toxic to bladder cancer cells than to peri‐tumor stroma cells.

### The iG2 suppressed the proliferation and enhanced the apoptosis of tumor cells

**Figure 2:**
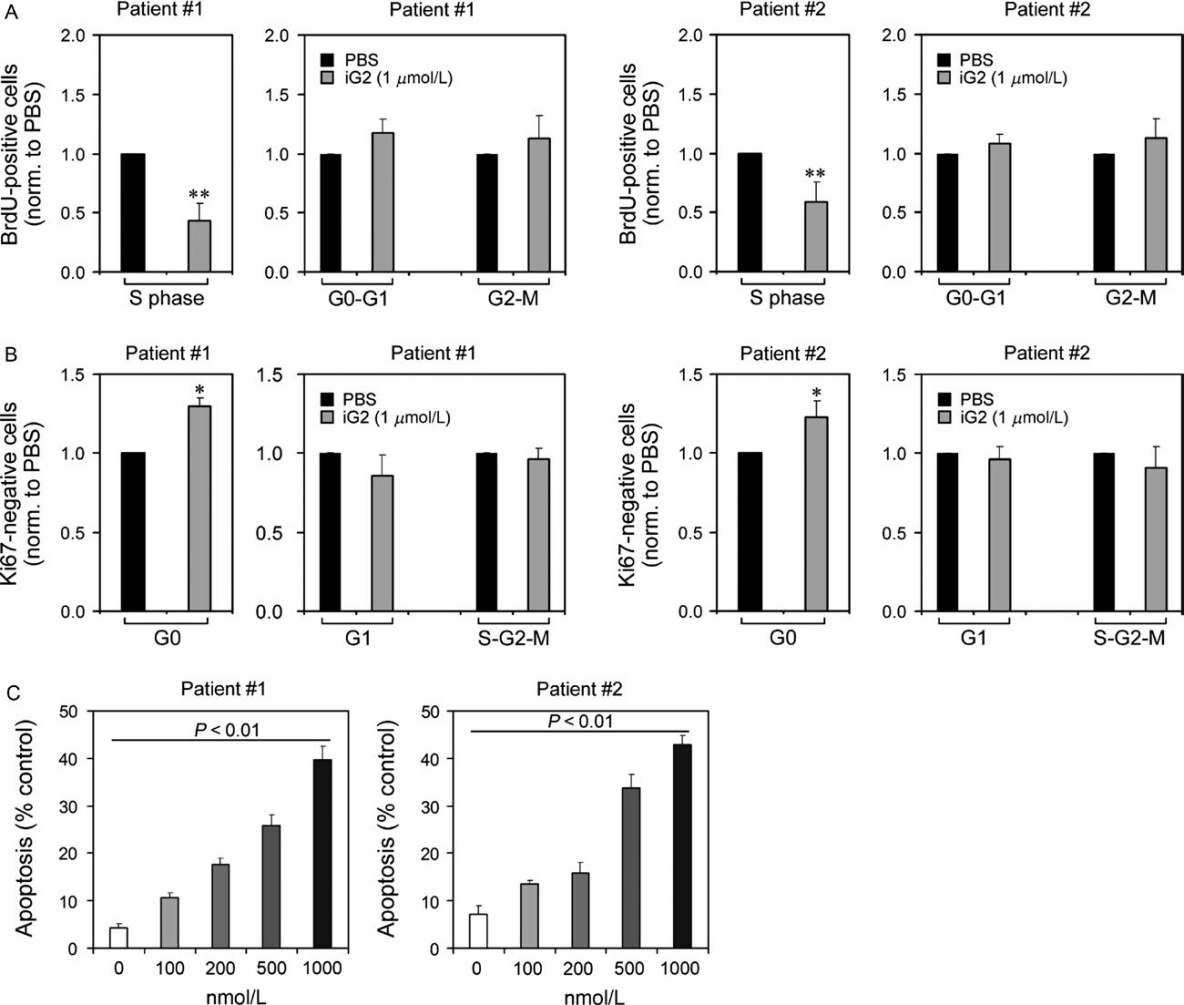
Attenuated proliferation and enhanced apoptosis upon iG2. (A–B) Cancer cells from patient 1# and 2# were treated with iG2 and the cell cycle was determined with BrdU staining (A) or Ki67 staining (B). (C) Cancer cells from patient #1 and #2 were treated with iG2 at indicated concentrations and the cell apoptosis was determined.

Whether iG2 affected cell proliferation and apoptosis of bladder cancer cells was studied. The results showed that the S phase of the cell cycle was reduced, whereas the G0 phase was enhanced by iG2 stimulation (Fig. 2A and B), indicating the proliferation of tumor cells could be attenuated by iG2. Meanwhile, the cell apoptosis of cancer cells was increased by iG2 stimulation in a dose‐dependent manner (Fig. 2C).

### The iG2 inhibited self‐renewal ability of tumor cells

**Figure 3:**
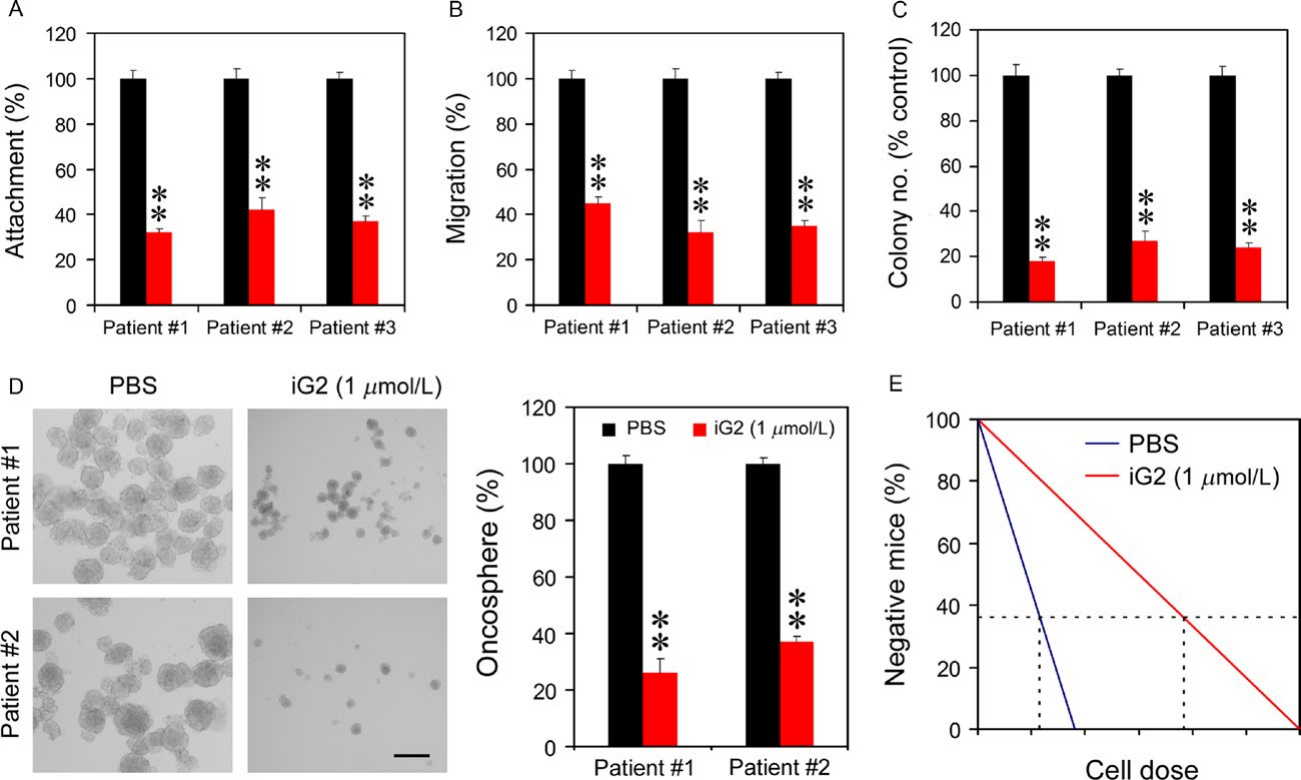
Impairment of cancer cell self‐renewal by iG2. (A) Reduction in cancer cell attachment to matrigel by iG2. (B) Cancer cell migration through transwell was suppressed by iG2. (C) Inhibition of cancer cell colony formation by iG2. (D) Oncosphere numbers were decreased by iG2 stimulation. (E) Tumor formation capability was attenuated by iG2.

CSCs are the primary cells that initiate tumor formation 7. The self‐renewal ability of bladder cancer cells with iG2 stimulation was investigated. When tumor cells were put into the dish coated with matrigel, iG2 reduced the attachment of cancer cells to the matrix (Fig. 3A). Meanwhile, the migration of the tumor cells through the transwell was attenuated by iG2 (Fig. 3B). In colony formation assay, iG2 decreased the colony number of tumor cells (Fig. 3C). Moreover, the oncosphere numbers of bladder cancer were reduced by iG2 too (Fig. 3D). Finally, the tumor formation by bladder cancer cells with serial dilution in mice was examined. As shown in Figure 3E, the iG2‐treated cells needed a higher cell number to form tumor in 40% mice when compared with the control (Fig. 3F). These results suggest that the self‐renewal ability of bladder cancer cells was mitigated by iG2.

### The iG2 decreased the tumor formation of bladder cancer cells

**Figure 4:**
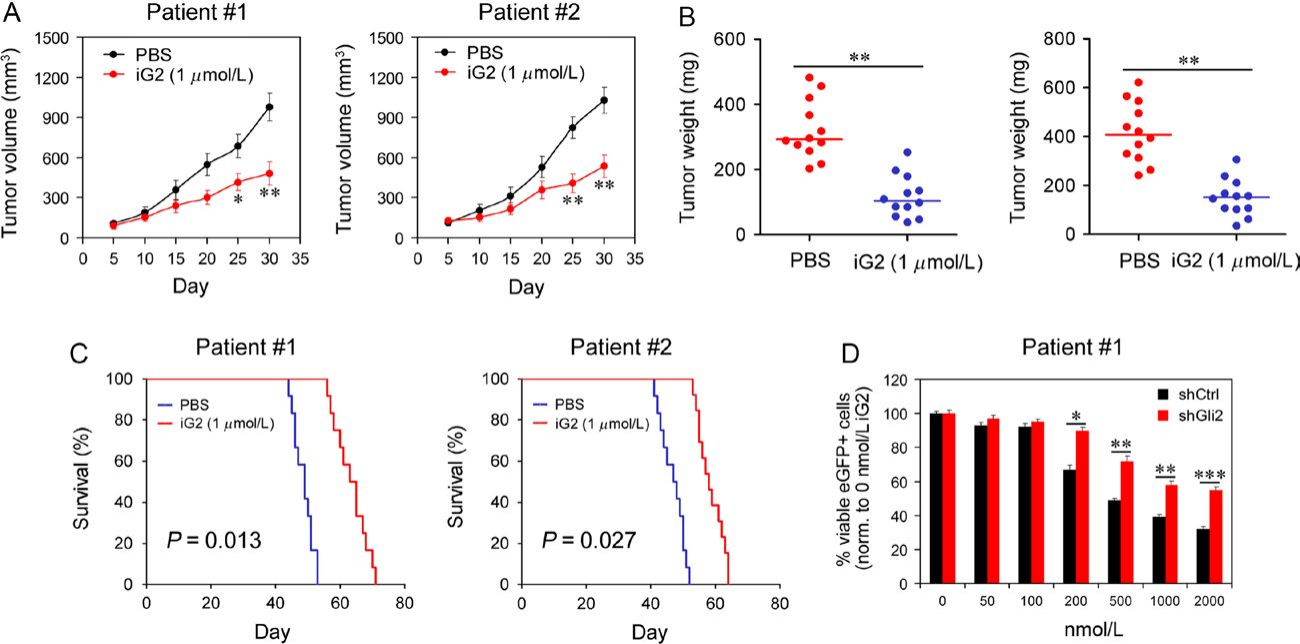
Tumor growth of bladder cancer cells was inhibited by iG2. (A–B) Tumor growth (A) and weight (B) were reduced by iG2 in vivo. Tumor cells from two patients were inoculated to nude mice (*n* = 12). Tumor growth was monitored and tumor weight was determined at day 30. (C) Survival of mice was increased by iG2 treatment. (D) Gli2 sensitized tumor to iG2 treatment. Tumor cells from patient 1# were transfected with shCtl and shGli2. Stable transfected cell lines were treated with gradient concentration of iG2 for 24 h. *P < 0.05, **P < 0.01.

Whether iG2 could be used as a therapeutic agent was tested in mouse model. As shown in Figure 4A and B, the tumor growth and tumor weight were dramatically decreased by iG2. However, the survival of mice was improved (Fig. 4C). To examine whether the therapeutic effect of iG2 correlated with the Gli2 activity in tumor cells, the expression of Gli2 in bladder cancer cells was knocked down. As shown in Figure 4D, after knock‐down of Gli2, the toxicity of Gli2 to tumor cells was largely attenuated, indicating that the inhibition of tumor growth by iG2 could be attributed to the inhibition of Gli2 activity.

## Discussion

In this study, we identified a novel Gli2 inhibitor iG2 from s*treptomyces roseofulvus*, which dramatically blocked tumorigenesis of bladder cancer cells. The iG2 can strongly downregulated the expression of Gli1, Gli2, and downstream Jag2 genes in bladder cancer cells. In accordance, iG2 reduced the proliferation and promoted the apoptosis of the tumor cells in vitro. The self‐renewal of bladder cancer cells was attenuated by iG2 treatment. Application of iG2 in mouse tumor model decreased tumor progression and enhanced the survival of mice. Therefore, iG2 is a potential unique chemotherapeutic drug for the treatment of bladder cancer in clinic.

The iG2 is a new Hh inhibitor, as a natural product separated from s*treptomyces roseofulvus*. Today, more than 80% of antibiotics are sourced from *Streptomyces* including penicillin and kanamycin 32. Very fortunately, we identified a novel product from *streptomyces roseoflavus subsp. Hangzhouensis n. subsp*. To explore the possible downstream signaling pathway activated by iG2, we found that iG2 dramatically down‐regulated Gli2 of Hh pathway in bladder cancer cells. So far, a few compounds and derivatives from natural products have been discovered as Hh inhibitors, such as cyclopamine 33. The detail of their working mechanism and their potential usage in clinical treatment of cancer need to be well evaluated in the future.

Our results first demonstrate that the self‐renewal of CSCs in bladder cancer cells can be targeted by iG2. The Hh pathway plays a central role in the maintenance of CSCs in a variety of human cancers including bladder cancer 2, 15, 27. Clinical trials with pathway antagonists have validated Hh signaling as a *bona fide* anticancer target in many cancers, but few in bladder cancer 15, 27. Here, our results suggest that the iG2 predominantly inhibits the proliferation and promotes the apoptosis of bladder cancer cells. The ability of attachment, migration, and colony formation of tumor cells are generally repressed by iG2. The stemness of CSCs evaluated by sphere‐formation assay and tumor formation assay with tumor cell serial dilution clearly showed that iG2 stimulation attenuated the self‐renewal of CSCs. In mouse model, application of iG2 decreased the tumor growth and enhanced the survival of mice. Because CSCs usually are hypothesized to be responsible for the emergence of drug resistance, disease progression, and distant metastasis 34. The combination of iG2 that target CSCs with standard treatments that target the bulk tumor population may prevent relapse, resistance, and receive durable responses in bladder cancer.

The iG2 may be used in tumor that resists to SMO inhibitors. Currently, most of Hh pathway antagonists in clinical testing are designed to target SMO, such as GDC‐0449, LDE225, and PF‐04449913 15, 27. Actually, both in clinic and experimental research, SMO resistance in tumor has been reported 28, 35. Even in some tumors, SMO‐independent aberrant Gli activation was observed 27. Therefore, the development of SMO downstream Gli inhibitors is strongly in need. The iG2 is ideal to be used in these tumors as a Gli2 inhibitor.

The iG2 may be safely used in the clinical treatment of cancer patients. In our animal model, iG2 was well tolerated in mice. In addition, the toxicity of iG2 to peri‐tumor cells was minimal when compared with that to the tumor cells. However, the activation of Hh pathway in peri‐tumor cells was equally inhibited as that in tumor cells. The inhibition of Hh pathway in tumor stromal cells may provide additional antitumor effects of iG2 in bladder cancer 36.

In conclusion, iG2 is a novel Gli2 inhibitor that is potential to be used to target self‐renewal of CSCs in bladder cancer patients. In the future, the molecular mechanism for the inhibition of Gli2 by iG2, the safety and efficacy of iG2 in clinic, and the combination usage of iG2 with other drugs need to be further studied.

## Conflict of Interest

The authors disclose no potential conflicts of interest.
